# Impact of worklist selection on point-of-care ultrasound workflow – a quality improvement project

**DOI:** 10.1186/s12913-025-12234-6

**Published:** 2025-01-14

**Authors:** Jonathan Rowland, Jessa Baker, Natassia Dunn, Matthew Whited, Soheil Saadat, J. Christian Fox

**Affiliations:** 1 Department of Emergency Medicine, University of California, Irvine, Orange, CA 92868 USA; 2 School of Medicine, University of California, Irvine, CA 92697 USA

**Keywords:** Ultrasound, Point-of-care ultrasound, Workflow, Quality improvement, Clinician satisfaction

## Abstract

**Background:**

Research demonstrates that Point-of-care ultrasound (POCUS) improves clinical outcomes for patients. Improving clinician satisfaction with POCUS should promote utilization into everyday practice, leading to improved clinical outcomes. Despite this benefit, there are still barriers to use including POCUS workflow. This project was undertaken to improve the functionality of the existing POCUS workflow and move toward an “encounter-based” system by automating worklist generation. It aimed to streamline the POCUS workflow, primarily determine if there was improved clinician satisfaction with the new workflow, and secondarily determine the change in revenue generation from decreased errors in data entry.

**Methods:**

A new workflow was created which automatically populated every registered Emergency Department (ED) patient into the worklist upon patient registration. Clinician feedback on their use of the new workflow was sought via survey after implementation. The number of medical record number (MRN) entry errors prior to and following implementation was manually reviewed and calculated.

**Results:**

There was a strong preference for the new workflow, with 36 of 38 (94.7%) clinicians finding it to be more convenient and 37 of 38 (97.4%) finding it to be preferable to use compared to the old workflow. Implementation also resulted in a 36% reduction in database studies containing an MRN data entry error.

**Conclusions:**

An “encounter-based” workflow is strongly preferred over manual data entry for POCUS workflow among clinicians. Additionally, there was no cost to the intervention and the total data entry errors were significantly reduced, allowing for improved quality review and increased revenue.

**Supplementary Information:**

The online version contains supplementary material available at 10.1186/s12913-025-12234-6.

## Background

Numerous stakeholders contribute to an efficient Point-of-Care Ultrasound (POCUS) program at a successful healthcare institution. These include patients, clinicians, medical directors, nurse managers, hospital administration, legal partners, biomedical engineering, information systems and technology, and coding and billing [1]. While a large multidisciplinary team is at play, the primary focus remains on patient-related outcomes and satisfaction. It has been shown repeatedly that POCUS can improve clinical outcomes through many different avenues, including increased safety and success of bedside procedures, individualized management of care, decreased length of Intensive Care Unit (ICU) stays, shortened time to initial treatment, return of spontaneous circulation (ROSC) after cardiac arrest, and increased overall survival [2–6]. Therefore, it stands to reason that improving clinician satisfaction with the use of POCUS should promote incorporation and utilization into everyday practice, which would likely result in further improved clinical outcomes for patients.

Though research demonstrates the positive impact POCUS has on patient outcomes, there are still several barriers to use. One of the main barriers includes POCUS workflow [1]. Many POCUS workflow systems involve manual data entry of patient information, which can be cumbersome and time-consuming for physicians to use. Previous studies have shown a connection between cognitive overload and increased time spent data gathering with increased medical errors [7]. New and improved clinical workflow systems that decrease cognitive load have shown evidence of improved clinician satisfaction and clinical outcomes [8]. Additionally, previous studies have demonstrated workflow compliance issues, specifically in documentation and billing [1, 9, 10]; when POCUS workflow is inconvenient, rates of documentation and billing are likely to decrease. With this in mind, this quality improvement (QI) project was undertaken to improve the functionality of the existing POCUS workflow and move toward a more “encounter-based” system by automating worklist generation. This project aimed to specifically, 1) streamline entering patient information into the ultrasound machines to improve efficiency and decrease errors in data entry, 2) allow images to be seen in the Electronic Medical Record (EMR) by all treating providers, 3) determine if there was improved clinician satisfaction with the new workflow system, and 4) determine change in revenue generation from decreased errors in data entry.

## Methods

This QI project was performed at a tertiary, level 1 trauma, academic Emergency Department (ED). The annual ED volume is approximately 65,000 patients per year, and the average number of POCUS scans per year is 16,400. Thirty-seven attending physicians, fellows, and nurse practitioners are credentialed in POCUS. Resident physicians, while not credentialed, also perform POCUS under direct observation by attendings and fellows who are credentialed in POCUS. The original POCUS workflow included manually entering a patient’s medical record number (MRN) into the touchscreen ultrasound machine, followed by manually entering the operator identification (ID). The clinician would then perform the appropriate scan of the patient and ‘end’ the scan to save the images and/or video clips. No manual EMR order was required. This system had been used for 20 years at this institution.

To create the modified POCUS workflow, the information technology (IT) department assisted in creating a method for automatically populating every registered ED patient into the worklist for all ED ultrasound machines. This was done by creating a temporary ED POCUS order, which was automatically generated by the EMR (EPIC, Epic Systems Corporation) once the patient was registered. This occurred instantaneously, including for resuscitation and trauma cases brought in by ambulance. Selecting a patient from the worklist allowed stored images to be automatically and appropriately archived within the patient’s chart. Thus, the images could almost immediately be reviewed by anyone with EPIC access via the Picture Archiving and Communications System (PACS) (AGFA HealthCare, in this case), the same way an x-ray or computed tomography (CT) image would be viewed. Patient information would reside on the POCUS worklist until midnight when the list would reset for the new date.

After this was implemented throughout the department, clinicians using POCUS were able to select the appropriate patient from the dropdown patient worklist, leaving only the operator ID to be manually entered. After the images were obtained and saved, this new system automatically linked the saved POCUS images to the patient’s chart in the EMR, allowing the images to be seen by all treating providers both during that encounter and in future encounters. This required no additional cost from department administration, did not require any middleware software, and took only three months from initial email to implementation. The clinician’s interpretation of the scan was documented in a separate procedure note.

Clinician feedback related to their use of the new POCUS workflow, as well as their overall satisfaction with the change, was the primary outcome. A short survey (Appendix A) was developed specifically for this study and was performed via electronic survey (Google Forms) four months after implementation of the system. A reminder email was sent three days after the initial email, a second reminder email was sent one week later, and a third and final reminder email was sent after one month. Participation in this survey was voluntary and anonymous. The survey included six questions, took an estimated 2 min or less, and respondents were given the opportunity to provide feedback using a free response comment box. Secondary outcomes included the number of MRN data entry errors prior to and following implementation, which was manually reviewed and calculated.

## Results

**Fig. 1:**
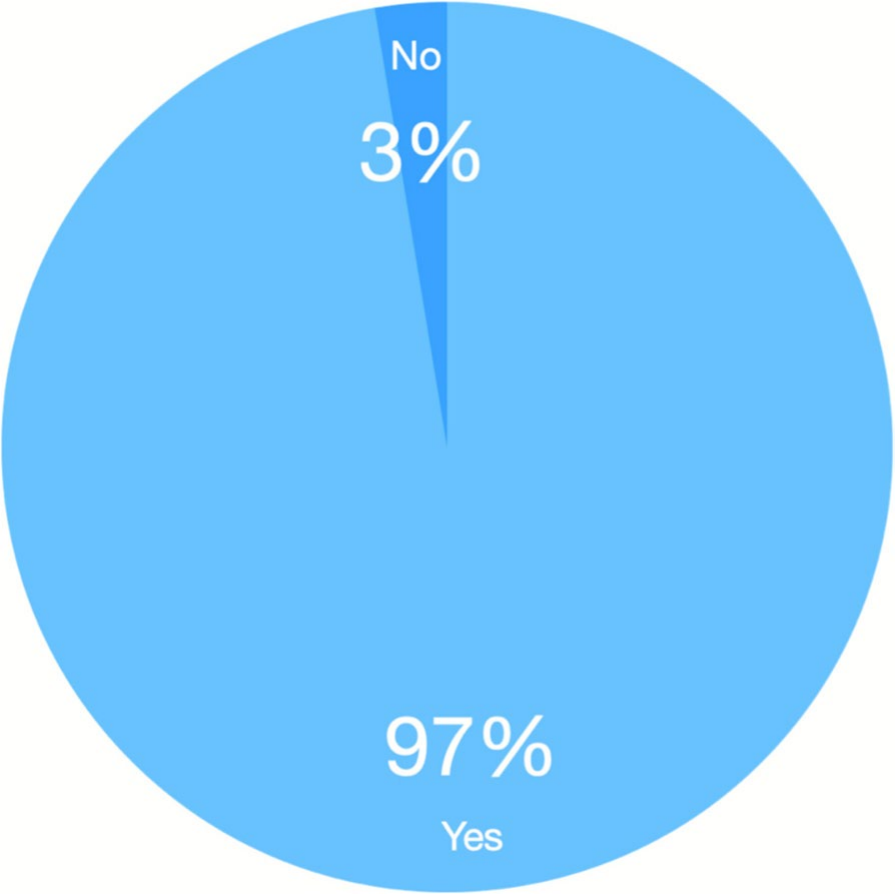
Clinician preference for new workflow

A total of 50 out of 69 (72.5%) clinicians completed the survey. Respondents were made up of 23 resident physicians years 1–3 (46%), 15 attending physicians (30%), 7 fellows (14%), and 5 nurse practitioners (10%). Thirty-eight of the 50 (76%) clinicians who completed the survey had tried using the new workflow during the four-month period of implementation. Among these users, there was a strong preference for the new workflow, with 36 of 38 (94.7%) finding it to be more convenient to use than the old workflow, and 37 of 38 (97.4%) finding it to be preferable to use compared to the old workflow (Fig. 1). The clinician who found the new workflow to be less convenient though still preferable had the following comment: *“The old [workflow] is easier because you type [the MRN] in, and sometimes scrolling through the list [with the new workflow] is annoying – but having the images saved to the patient’s chart is worth the annoyance.”*

Nine of the 38 users commented that the worklist did not function consistently, prompting them to revert to manually entering the patient’s information. Two of the 38 users commented that the automated worklist was too long to scroll through.

**Fig. 2:**
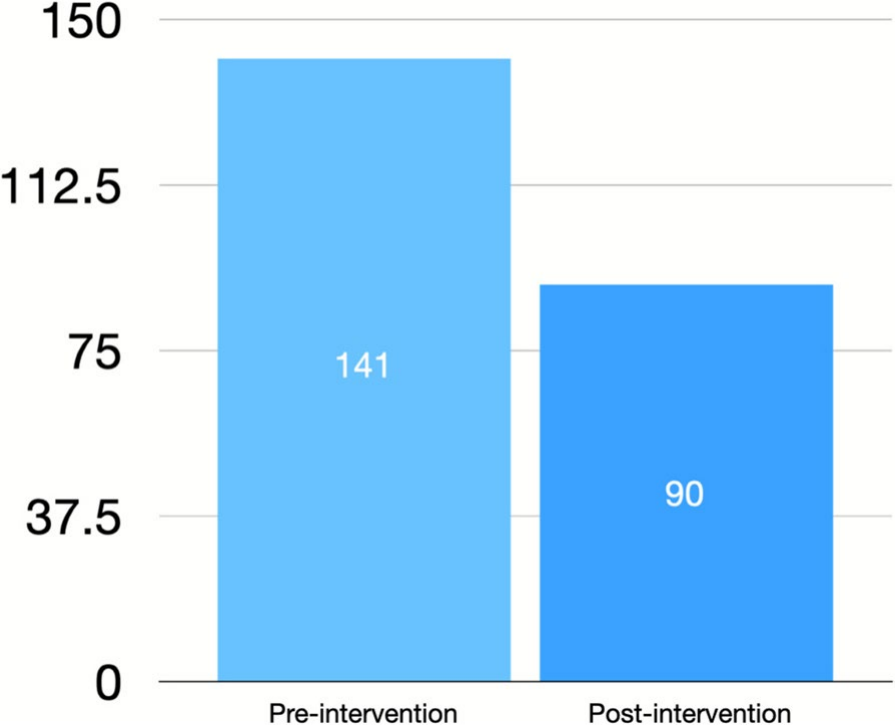
MRN data entry errors pre- and post-intervention resulting in studies not billed for

Through manual review, there were 141 studies that had MRN data entry errors in the 138-day period before implementation of the new POCUS workflow. These errors included adding additional numbers to the 7-digit MRN, not including enough numbers, or using the incorrect number. Due to the data entry errors, the studies were unable to be linked to a patient encounter and, therefore, were not billed for and were not assessed during the quality review process. In the 138-day period following implementation, there were only 90 MRN data entry errors (Fig. 2).^1^ This resulted in a relative 36% reduction in database studies containing an MRN data entry error, which led to a revenue gain of approximately $1,800 in professional charges and $5,600 in technical charges. In theory, with ongoing and streamlined use of automated worklist generation, there should eventually be very few data entry errors resulting in successful billing and quality review of nearly all studies performed.

## Discussion

Automatic worklist generation and selection, or “encounter-based” workflow, is strongly preferred over manual data entry for POCUS workflow among clinicians. Similar studies performed have demonstrated the positive effects of implementing an automated POCUS workflow, such as increased POCUS utilization [11] and significant increases in compliance with POCUS documentation and image archiving, thus leading to increased billing and revenue generation [11–13]. Our study differs in that clinician satisfaction was the primary outcome of this study. A strong majority found the new workflow to be more convenient to use and preferable over the old workflow. It is reasonable to expect that increased clinician satisfaction with POCUS use should lead to increased utilization and, therefore, improved clinical outcomes. Additionally, using the new workflow allowed the images to be viewed in real-time in the EMR by all treating providers, promoting expeditious multi-disciplinary management of emergent pathology such as ruptured ectopic pregnancies.

Finally, the total number of data entry errors related to patient MRN was significantly reduced using an automated worklist. Such errors limit our ability to enter the patient’s chart for quality review of the acquired POCUS images, and therefore a reduction in errors allowed for improved quality review. In addition, these errors limit our ability to appropriately bill for these studies, resulting in a loss of revenue; the reduction in errors using the new workflow allowed for over $7,000 in increased revenue generation during the examined post-implementation period.

The 90 MRN data entry errors seen post-implementation of the new workflow were all due to manual data entry errors. There are several reasons why some users continued to enter patient information manually rather than use the automated worklist. The ultrasound machines are only able to display the worklist for a single date; providers using the ultrasound machines shortly after midnight had to manually change the worklist date to find patients that had checked in prior to 12:00 am. If the date had been manually changed on the machine by a previous provider, then patients checking in after 12:00 am were not viewable on the automated worklist unless the date was manually changed again. Therefore, providers using the machines overnight may have had difficulty finding their patients due to the date change. Additionally, the machines were used throughout a large, 50 + bed ED; on occasion, the machines would lose connectivity to the Wi-Fi when being transported, making the automated worklist no longer viewable. Many users were unaware of how to reconnect the machines to the Wi-Fi. Furthermore, each patient only populated to the worklist a single time; when a study was ‘ended’ after performing a scan, the patient no longer appeared on the worklist as the infrastructure was not set up to complete subsequent exams. Therefore, if a repeat scan was performed, the patient could not be selected from the worklist. Lastly, one of the two users who commented on the length of the worklist requested that the worklist be automatically alphabetized. The worklist was organized by patient arrival time; however, it could be sorted by last name, which required an extra ‘click’ on the ultrasound touchscreen. It may be that some users were unaware of this extra step required to sort the list alphabetically; therefore, rather than scrolling through the list, manual data entry was used.

There are several limitations to this study. This QI project examined the effects of a single method of streamlining POCUS workflow. There are other methods that could additionally be examined and compared, such as order-based workflows (versus our encounter-based workflow) or using barcode scanners to attach saved POCUS images to patient charts. Our department at the time of implementation of the new workflow did not use any middleware software, however this workflow option should still suffice for other departments that do utilize a middleware solution, and some middleware options may further streamline the workflow process. While there was no additional cost to implement our automated workflow, this may also vary depending on the EMR and on the capabilities of the individual IT department. Additionally, documentation and billing are separate processes from the new workflow; our study did not examine the effects of this change in workflow on compliance with documentation, nor did it examine rates of POCUS use prior to and following implementation. Lastly, this study looked at the relative immediate effects of the implementation of a new workflow after several months; however, it did not examine the long-term effects on clinician satisfaction and data entry errors.

## Conclusion

Based on this study, we suggest moving toward an encounter-based workflow and eliminating manual data entry in POCUS workflow. Benefits include happier clinician users, no cost for implementation, improved billing/revenue, improved quality review, and the technical flexibility to easily communicate with the EMR when the data is appropriately and fully connected, which was preferred among our IT department. Future studies should consider measuring changes in efficiency as well as objective effects on clinical outcomes following implementation of workflow optimization.

## Supplementary Information

Supplementary Material 1.

Supplementary Material 2.

## Data Availability

All data generated or analyzed during this study are included in this published article and its supplementary information files.

## Footnotes

1 Following implementation, there was a transition period in which many clinicians were still manually entering data rather than using the new workflow. Additionally, if the new workflow failed for any reason (for example, failure of machine Wi-Fi), it was advised to revert to the old workflow and manually enter patient data.

## References

[1] Saati A, Au A, Chu T, Davis RL, Singla R, Smith J, White JL, Lewiss RE. Creating an Efficient Point-of-Care Ultrasound Workflow. J Point Care Ultrasound. 2020;5(2):31–2. 10.24908/pocus.v5i2.14429. (Published 2020 Nov 18). 10.24908/pocus.v5i2.14429 PMC9979953 36896441

[2] Chen Z, Hong Y, Dai J, Xing L. Incorporation of point-of-care ultrasound into morning round is associated with improvement in clinical outcomes in critically ill patients with sepsis. J Clin Anesth. 2018;48:62–6. 10.1016/j.jclinane.2018.05.010. (Published 2018 May 12). 29763777 10.1016/j.jclinane.2018.05.010

[3] Zieleskiewicz L, Lopez A, Hraiech S, et al. Bedside POCUS during ward emergencies is associated with improved diagnosis and outcome: an observational, prospective, controlled study. Crit Care. 2021;25(1):34. 10.1186/s13054-021-03466-z. (Published 2021 Jan 22). 33482873 10.1186/s13054-021-03466-z PMC7825196

[4] Atkinson PR, Beckett N, French J, Banerjee A, Fraser J, Lewis D. Does Point-of-care Ultrasound Use Impact Resuscitation Length, Rates of Intervention, and Clinical Outcomes During Cardiac Arrest? A Study from the Sonography in Hypotension and Cardiac Arrest in the Emergency Department (SHoC-ED) Investigators. Cureus. 2019;11(4):e4456. 10.7759/cureus.4456. (Published 2019 Apr 13). 31205842 10.7759/cureus.4456 PMC6561518

[5] Shrestha GS, Srinivasan S. Role of Point-of-Care Ultrasonography for the Management of Sepsis and Septic Shock. Rev Recent Clin Trials. 2018;13(4):243–51. 10.2174/1574887113666180412165405. 29651944 10.2174/1574887113666180412165405

[6] Thom C, Ahmed A, Kongkatong M, Moak J. Point-of-care hip ultrasound leads to expedited results in emergency department patients with suspected septic arthritis. J Am Coll Emerg Physicians Open. 2020;1(4):512–20. 10.1002/emp2.12167. (Published 2020 Jun 30). 33000078 10.1002/emp2.12167 PMC7493574

[7] Khairat SS, Dukkipati A, Lauria HA, Bice T, Travers D, Carson SS. The Impact of Visualization Dashboards on Quality of Care and Clinician Satisfaction: Integrative Literature Review. JMIR Hum Factors. 2018;5(2):e22. 10.2196/humanfactors.9328. (Published 2018 May 31). 29853440 10.2196/humanfactors.9328 PMC6002673

[8] McGrath SP, Perreard IM, Garland MD, Converse KA, Mackenzie TA. Improving Patient Safety and Clinician Workflow in the General Care Setting With Enhanced Surveillance Monitoring. IEEE J Biomed Health Inform. 2019;23(2):857–66. 10.1109/JBHI.2018.2834863. (Published 2019 May 9). 29993903 10.1109/JBHI.2018.2834863

[9] Lahham S, Moeller J, Kurzweil A, Choi H, Saadat S, Dang E, Mazumder P. Evaluation of Adherence to Emergency Department Point-of-Care Ultrasound Documentation and Billing Following Intervention. J Med Ultrasound. 2022;30(3):211–4. 10.4103/jmu.jmu_76_21. (Published 2022 April 15). 36484038 10.4103/jmu.jmu_76_21 PMC9724462

[10] Baker J, Greb A, Rowland J, Whited M, Saadat S, Fox JC. Examining the Durability of an Inexpensive Intervention for Improving Point-of-care Ultrasound Documentation Rates. J Med Ultrasound Published. 10.4103/jmu.jmu_102_23. (Published 2024 September 20). 10.4103/jmu.jmu_102_23s PMC12161696 40521320

[11] Rong K, Chimileski B, Kaloudis P, Herbst MK. Impact of an epic-integrated point-of-care ultrasound workflow on ultrasound performance, compliance, and potential revenue. Am J Emerg Med. 2021;49:233–9. 10.1016/j.ajem.2021.06.009. (Epub 2021 Jun 7). 34146922 10.1016/j.ajem.2021.06.009

[12] Thompson B, Schoenfeld E, Westafer L, Visintainer P, Budhram G. Implementation of an automated, user-centered point-of-care ultrasound workflow improves documentation and billing. Acad Emerg Med. 2023;30(3):180–6. 10.1111/acem.14654. (Epub 2023 Jan 27). 36617844 10.1111/acem.14654

[13] Flannigan M, Adhikari S. Point-of-Care Ultrasound Work Flow Innovation: Impact on Documentation and Billing. J Ultrasound Med. 2017;36(12):2467–74. 10.1002/jum.14284. (Epub 2017 Jun 24). 28646595 10.1002/jum.14284

