# Supplementary material for: Impact of worklist selection on point-of-care ultrasound workflow – a quality improvement project
Source: BMC Health Serv Res. 2025 Jan 14;25:76. doi: 10.1186/s12913-025-12234-6 (PMC11730821; doi:10.1186/s12913-025-12234-6)
Supplement: Supplementary file 1 — Supplementary Material 1. [file 12913_2025_12234_MOESM1_ESM.docx]

**Appendix A:**

New Ultrasound Workflow Feedback Survey

**response required*

1. Have you used the new ultrasound workflow? (i.e. selecting patient from worklist button) *

1. Yes
2. No

*If yes, what did you think?*

2. Assuming all machines and software are appropriately functioning, which workflow is more convenient? *

1. Old workflow (manually typing MRN)
2. New workflow (select patient from worklist)
3. Other: _______

3. Assuming all machines and software are appropriately functioning, which workflow would you prefer to use? *

1. Old workflow (manually typing MRN)
2. New workflow (select patient from worklist)
3. Other: _______

4. Any feedback at all about ultrasound workflow? (OPTIONAL)

(Free response space)

*Demographics*

5. Who are you? *

1. R1
2. R2
3. R3
4. Fellow
5. Attending
6. NP

6. How often do you perform POCUS on shift?

1. Never
2. Some shifts
3. Most shifts
4. Every shift
5. Multiple times every shift

*Survey administered via Google Forms*
